# Editorial: Biofouling, biocorrosion and biodeterioration: Recent advancements

**DOI:** 10.3389/fbioe.2023.1144671

**Published:** 2023-02-10

**Authors:** Ioannis A. Kartsonakis, Viswanathan S. Saji, Leto-Aikaterini Tziveleka, Raman Singh, Daniel John Blackwood, Tingyue Gu

**Affiliations:** ^1^ Physical Chemistry Department, School of Chemical Engineering, National Technical University of Athens, Zographos, Attiki, Greece; ^2^ Interdisciplinary Research Center for Advanced Materials, King Fahd University of Petroleum and Minerals (KFUPM), Dhahran, Saudi Arabia; ^3^ National and Kapodistrian University of Athens, Department of Pharmacy, Section of Pharmacognosy and Chemistry of Natural Products, Athens, Greece; ^4^ Department of Mechanical and Aerospace Engineering, Department of Chemical and Biological Engineering, Monash University—Clayton Campus (Melbourne), Melbourne, VIC, Australia; ^5^ Department of Materials Science and Engineering, National University of Singapore, Singapore, Singapore; ^6^ Department of Chemical and Biomolecular Engineering, Ohio University, Athens, OH, United States

**Keywords:** biofouling, biocorrosion, biodeterioration, preventive approaches, applications, bioelectrochemistry

The Research Topic *Biofouling, Biocorrosion and Biodeterioration: Recent Advancements* of the Frontiers in Bioengineering and Biotechnology, aimed to present interdisciplinary contributions emphasizing recent developments in this important Research Topic. Biofouling, biocorrosion and biodeterioration are directly related to the presence of microorganisms and the formation of biofilms. Various advances have been made in recent years in this economically important and scientifically challenging research area. The biofilm development in service conditions influences the interplay between a solid material’s surface and the environment, often leading to accelerated materials deterioration. Strategies typically employed to control biofouling, biocorrosion and biodeterioration include physical scrubbing, biocides and corrosion inhibitors, beneficial biofilms, cathodic protection, surface modifications (including novel antimicrobial surfaces) and protective coatings.

This Research Topic comprises six papers from eminent researchers worldwide, including Australia, China, Chile, Mexico, Saudi Arabia, Thailand, United Kingdom and United States. A wide range of Research Topic is covered, including corrosion protection of oil and gas equipment using corrosion inhibitors and biocides against microbial deposition (Suarez et al.; Tuck et al.; Salgar-Chaparro et al.), development of bioactive materials for tissue engineering (Zurita-Méndez et al.), growth of enzymes-producing bacteria as a function of pH (Espinoza-Vergara et al.) and the impact of microbiologically influenced corrosion (MIC) on mechanical properties of pipeline steels (Li et al.). All contributions to this Research Topic focus on one or more of the aforementioned highlighted research areas.

Three articles in this Research Topic present studies on corrosion inhibitors/biocides that react against microbial deposition to protect steel in industrial systems. The article by Suarez et al. highlights the importance of inhibiting under-deposit microbial corrosion, which was investigated in real-time using a multi-electrode array and surface profilometry analyses. The presence of microbes deteriorated corrosion protective films deposited on steel pipeline and equipment surfaces in industrial service conditions such as those in oil and gas production. Their results provided interesting observations on the efficiency of corrosion inhibitors in complex environments involving deposits and microorganisms.

The research article by Tuck et al. sheds light on the biocidal activity of the organic corrosion inhibitor, CTA-4OHcinn, against mature biofilms in simulated marine conditions. The biocidal activity was investigated against mature, multi-species biofilms developed from oilfield samples on carbon steel over two weeks. The reduced cell numbers and total adenosine triphosphate content demonstrated CTA-4OHcinn’s biocidal activity. Moreover, biofilm compactness, live cell quantity and viability were significantly reduced after the biocide application, as evidenced by confocal microscopy and post-image analysis. Additionally, no structures attributed to complex, multi-species biofilm were present in biofilms after CTA-4OHcinn treatment, while CTA-4OHcinn was found to target the bacterial cell membrane resulting in lysis. It was found that the biocide can be considered a multi-functional compound for use in industrial systems such as pipelines, effective at inhibiting corrosion and limiting early and mature biofilm formation.

The research article by Salgar-Chaparro et al. demonstrated that the extent of MIC is highly dependent on the characteristics of the environment, thus contributing to a better understanding of the involvement of versatile microorganisms in biofilms formed on industrial assets. AISI 1030 carbon steel was used as test material for the MIC evaluation, while the corrosion behaviour of *Shewanella chilikensis* DC57, a bacterium isolated from an oilfield corrosion failure, was assessed under nitrate- and thiosulfate-reducing conditions. Their results proved that the evaluation of the MIC risk must consider both the microbial species present in a system as well as the environmental conditions, such as nutrient availability, and associated metabolic activities that microorganisms can perform on metallic surfaces under such conditions.

Concerning the development of bioactive materials for tissue engineering applications Zurita-Méndez et al. conducted experiments using materials based on bioactive glass particles, hyaluronic acid, collagen and polycaprolactone. Once in the form of scaffolds, they were evaluated for their mechanical properties, while, as coatings, they were evaluated using electrochemical techniques. Scaffolds composed of higher levels of bioactive glass particles showed minor compression strength, while the ones with higher levels of collagen/hyaluronic acid displayed higher compression strength and biocompatibility. Their work illustrated a corrosion mechanism governed by activation and finite diffusion through the porous layer.

The paper by Espinoza-Vergara et al. investigated bacterial growth of the catalase-producing *Pseudomonas aeruginosa* as a function of pH. Catalase is a scavenging enzyme involved in decomposing acutely toxic H_2_O_2_ to O_2_, contributing to the bacteria’s survival mechanism. Electrochemical techniques were used to monitor the enzymatic activity. Their results at pH 7 were correlated to the enzymatic conversion of H_2_O_2_ to O_2_, suggesting that catalase’s action was favored at neutral pH. The influence of pH on the adhesion of *P. aeruginosa* on aluminum alloy surfaces (AA2024 and AA6063) was further evaluated by employing atomic force microscopy. The most robust adhesion was found to occur at pH 6 on the surface of AA-6063, and it was attributed to the positive surface charge that this alloy exerts, reducing the repulsive electrostatic energy barrier and promoting cell adhesion.

The article by Li et al. addresses the impact of MIC on the degradation of mechanical properties of X80 carbon steel, an sub-area of MIC that deserves more attention, in view that the current MIC research focuses on pinhole leaks rather than catastrophic structural failures. Specifically, the microorganism employed was the sulfate-reducing bacterium (SRB), *Desulfovibrio vulgaris*, while variations in MIC severity were achieved by varying the headspace volumes in anaerobic bottles for SRB incubation. An increased headspace volume allowed more biogenic H_2_S to escape to the headspace, which reduced its cytotoxicity leading to better SRB growth. Corrosion pit depths and weight loss were more severe at a higher sessile cell count. It was found that SRB MIC made the X80 pipeline steel more brittle, as reflected by up to 23% ultimate strain loss in only 14 days.

In summary, this Research Topic demonstrates several novel developments that could benefit further advancements in this important research area.

